# Celecoxib vs diclofenac sodium in patients with knee osteoarthritis: A protocol for systematic review and meta analysis

**DOI:** 10.1097/MD.0000000000019680

**Published:** 2020-04-10

**Authors:** Hetao Huang, Jianke Pan, Weiyi Yang, Hongyun Chen, Guihong Liang, Lingfeng Zeng, Jun Liu, Biqi Pan

**Affiliations:** a Second School of Clinical Medicine, Guangzhou University of Chinese Medicine; b Department of Orthopaedics, Second Affiliated Hospital of Guangzhou University of Chinese Medicine, Guangdong Provincial Hospital of Chinese Medicine; c Department of Traditional Chinese Medicine, GuangDong Women and Children Hospital, China.

**Keywords:** celecoxib, diclofenac sodium, knee osteoarthritis, meta-analysis

## Abstract

**Background:**

Knee osteoarthritis (KOA) is one of the most common chronic muscular diseases in old people. The purpose of this meta-analysis was to compare celecoxib and diclofenac sodium in patients with knee osteoarthritis (KOA).

**Methods:**

Randomized controlled trials (RCTs) and clinical controlled trials (CCTs) comparing the use of celecoxib and diclofenac sodium in KOA patients were retrieved from each database from the date of database inception to September 2019. The outcome measurements were the treatment effect, visual analog scale (VAS) score, erythrocyte sedimentation rate (ESR), C-reactive protein (CRP) level, and complication rate. The pooled data were evaluated with Review Manager 5.3.5.

**Results:**

The literature will provide a high-quality analysis of the current evidence supporting celecoxib for KOA based on various comprehensive assessments including the treatment effect, visual analog scale (VAS) score, erythrocyte sedimentation rate (ESR), C-reactive protein (CRP) level, and complication rate.

**Conclusion:**

This proposed systematic review will provide up-to-date evidence to assess the effect of celecoxib in the treatment for patients with KOA.

**Research Registry registration number:**

reviewregistry827.

## Abstract 2

Supplemental Digital Content is available in the text

## 1 Introduction

Knee osteoarthritis (KOA) is one of the most common chronic progressive diseases in old people.^[1]^ The main manifestations of KOA are pain and dysfunction in the knees, which affect quality of life and lead to a high rate of disability in elderly individuals. KOA is one of the most common causes of joint pain and loss of motor function among middle-aged and elderly people in the United States.^2^,^3^ The approximate prevalence of KOA in the general global population is 12% to 35%.^[4]^ KOA has increased medical care expenditures and attracted much government attention in some Asian countries.^[5]^ Oral anti-inflammatory drugs (NSAIDs), physiotherapy, topical anti-inflammatory gels, and intra-articular injections are currently routine treatments for patients with KOA.^[6]^

The main objectives in the management of KOA have been to alleviate pain, restore function, slow down the progression of disease and maintain a patients health-related quality of life.^[7]^ For most clinicians, celecoxib and diclofenac sodium are the most commonly used NSAIDs for the treatment of KOA because of their effectiveness in the treatment of KOA. Many researchers have studied the efficacy and safety of celecoxib and diclofenac sodium in the treatment of KOA. However, due to the small sample size and various biases in individual studies, it is impossible to draw a definite conclusion. In our study, we searched the authoritative databases for clinical controlled studies of the use of celecoxib and diclofenac sodium in the treatment of KOA and conducted a systematic evaluation and meta-analysis strictly according to the Cochrane method. This study was performed to evaluate the efficacy and safety of celecoxib and diclofenac sodium in the treatment of KOA and to compare which has more advantages. Ultimately, this study provides high-level evidence-based medical guidance to clinicians as a reference for treatment decision-making.

## 2 Methods

### 2.1 Data sources and search strategy

The study was approved by the ethics committee of Guangdong Provincial Hospital of Chinese Medicine. We will adhere to the Preferred Reporting Items for Systematic Reviews and Meta-analysis (PRISMA) statements for reporting systematic reviews. Seven databases, including PubMed, EMBASE, Cochrane Central Register of Controlled Trials, China National Knowledge Infrastructure, Chinese Scientific Journal Database, Wanfang Data and Chinese Biomedical Literature Database, were investigated from their inception through January 2020. The reference lists of retrieved papers were also studied. The following search terms were used individually or in combination. The mesh terms in this paper are as follows:“celecoxib”, “diclofenac sodium”, “knee”, “osteoarthritis,” and “arthritis”. To increase the search range, no date and no language limits were imposed. Additionally, no restrictions on population characteristics were imposed. The specific search strategies for PubMed is shown in the Supplemental Table.

### 2.2 Inclusion criteria and study selection

#### 2.2.1 Participants

Only published articles enrolling adult participants with a diagnosis of KOA will be included. The patients gender, age, and grades of KOA will not be limited.

#### 2.2.2 Interventions

The intervention group will have treated with celecoxib.

#### 2.2.3 Comparisons

The control group will have received diclofenac alone.

#### 2.2.4 Outcomes

The primary outcomes of this meta-analysis were “the treatment effect”, and the secondary outcomes were “VAS scores”, “ESR”, “CRP”, and “complication rate”.

#### 2.2.5 Study design

**Figure 1:**
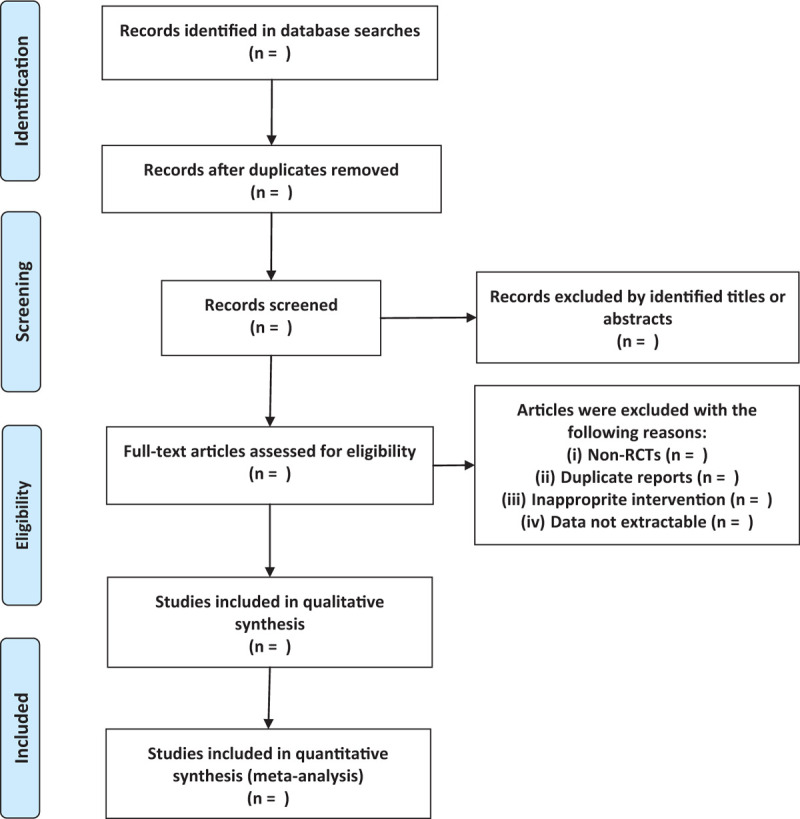
Flow diagram of study selection.

RCTs will be considered eligible for our study. Articles will be excluded if they are case reports, letters, editorials, and nonhuman studies. The flow diagram of the study selection is shown in Figure 1.

### 2.3 Data extraction

Data extraction included the first authors name, year of publication, sample size, diagnostic criteria, age, and sex of the participants, details of the intervention and control conditions, treatment duration, and outcome measurements for each study. Two authors (HTH, BQP) independently conducted the data extraction according to predefined criteria. Any uncertainty was resolved through discussion with another author (JL). The reasons for exclusion were recorded. The data were extracted from the included RCTs to a predefined Excel table (Microsoft Corp, Redmond, WA) and cross-checked by the 2 reviewers (HTH, JKP). In the event of missing data, we will attempt to contact the corresponding authors for details.

### 2.4 Assessment of methodologic quality

Two authors (HTH, WYY) independently assessed the methodological quality of each trial according to the standards advised by the Cochrane Handbook.^[8]^ Disagreements, if any, were resolved by discussion and reached consensus through a third reviewer (JL). The risk of bias was evaluated for each study by assessing the randomization process, the treatment allocation concealment, blinding of participants and personnel, blinding of outcome assessment, the completeness of the data, the reporting of results and other biases. Selective reporting bias was judged according to the published protocols for the registered clinical trials that were contained on the Chinese clinical trial registry (http://www.chictr.org) and international clinical trial registry of the US National Institutes of Health (http://clinicaltrials.gov) websites. We compared the outcome measures between the study protocol and the final published trial.

### 2.5 Data analysis

Review Manager 5.3.5 software (Cochrane Collaboration, Oxford, UK) was used for bias risk assessment, and Stata 14.0 was used for the statistical analysis of the data. Continuous data for the meta-analysis were analyzed and are expressed as the standard mean differences (SMDs) with 95% confidence intervals (CIs), and dichotomous data in the meta-analysis are presented as odds ratios (ORs) with 95% CIs. Heterogeneity among the studies was estimated with the I^2^ statistic. If the *I* ^2^ statistic was 50% or higher, indicating the presence of heterogeneity, a random-effects model was used; otherwise, a fixed-effects model was used. *P* values <.05 indicated statistical significance in all the results.

### 2.6 GRADE the evidence

The GRADE system was used to evaluate the quality of the evidence for each outcome. GRADE-pro GDT Online Tools (available on https://gradepro.org/) were used to evaluate the evidence regarding the included outcomes. Initially, RCTs were considered to be of high confidence in estimating an effect, and observational studies were considered to be of low confidence in estimating an effect. The reasons that may decrease the level of confidence included risk of bias, inconsistency, indirectness, imprecision, and publication bias. The reasons that may increase the level of confidence included a large effect, dose response, and accounting for all plausible residual confounding and bias. The GRADE evidence was divided into the following categories:

1. High-quality evidence, which indicated that further research was unlikely to change the confidence in the estimate of the effect;
2. Moderate-quality evidence, which indicated that further research was likely to have an important impact on the confidence in the estimate of the effect and may change the estimate;
3. Low-quality evidence, which indicated that further research was likely to have an important impact on confidence in the estimate of the effect and was likely to change the estimate; and
4. Very low-quality evidence, which indicated that we were very uncertain about the results.

## 3 Discussion

KOA is one of the most common chronic progressive diseases in the world.^[9]^ If early-stage KOA is not controlled satisfactorily, it will gradually develop into end-stage KOA, which is one of the main causes of disability in the elderly population.^[10]^ At present, there are many studies on the pathological mechanism of KOA, but the specific pathogenesis of KOA remains unclear.^11^,^12^,^13^,^14^,^15^,^16^ With the aging of the population, KOA will impose a very large economic burden on the global society.^17^,^18^,^19^

The standard treatment for KOA includes surgical treatment and nonsurgical treatment. Surgical treatment can be broadly classified as either joint-preserving or joint-replacing procedures. The purpose of nonsurgical treatment is patient education, pain control, delaying the progression of the disease, and improving function.^[20]^ NSAIDs are the most commonly used basic nonsurgical treatment for KOA; they have a good anti-inflammatory effect and can relieve pain. NSAIDs are prescribed when the patient presents with a swollen knee and exacerbation of pain. These agents act by blocking proinflammatory agents, such as prostaglandins and leukotrienes, by reversibly blocking the cyclooxygenase and lipoxygenase pathways. Selective COX2 inhibitors have an anti-inflammatory effect but cause many adverse reactions.^21^,^22^,^23^,^24^ Refecoxib was withdrawn from the market in 2004 due to its cardiovascular toxicity and clinically significant gastrointestinal events.^25^,^26^ However, celecoxib and diclofenac sodium are among the most common drugs prescribed for KOA among all NSAID drugs. Most previous studies have shown that celecoxib is an effective alternative treatment for the long-term relief of knee pain and improved joint function in KOA patients. However, previous conclusions were reached on the basis of independent research.

As the systematic review is based on the secondary research of published literature, there are undeniable methodological defects. In addition, the quality of the included studies determines the quality level and reliability of the final results. We will begin to conduct the review when the necessary trials are met, and all operating procedures will be performed in accordance of Cochrane Handbook to ensure that the provided information is helpful for clinicians and patients. This study is registered with the Research Registry and the unique identifying number is: reviewregistry827 (https://www.researchregistry.com/register-now#registryofsystematicreviewsmeta-analyses/registryofsystematicreviewsmeta-analysesdetails/5e4f4bab4db7810015c86b6d/).

## Acknowledgments

We would like to thank Professor Holger Schulenemann, Chairman of GRADE Working Group, Department of Clinical Epidemiology and Biomedical Statistics, McMaster University, Canada; Professor Li Youping, Director of Cochrane Center in China; Professor Yang Kehu, Director of GRADE Center in China; Professor Tian Jinhui, Evidence-based Medicine Center of Lanzhou University for their training on Cochrane system evaluation and grade system knowledge.

## Author contributions

**Conceptualization:** Hetao Huang, Jun Liu, Biqi Pan.

**Data curation:** Hetao Huang, Jianke Pan, Biqi Pan.

**Formal analysis:** Hetao Huang, Weiyi Yang, Biqi Pan.

**Funding acquisition:** Hongyun Chen, Guihong Liang, Lingfeng Zeng.

**Investigation:** Hetao Huang, Jun Liu.

**Methodology:** Guihong Liang, Lingfeng Zeng.

**Project administration:** Hetao Huang, Biqi Pan.

**Resources:** Weiyi Yang, Hongyun Chen.

**Software:** Hetao Huang, Jianke Pan, Biqi Pan.

**Supervision:** Jianke Pan.

**Validation:** Weiyi Yang, Hongyun Chen.

**Visualization:** Jianke Pan.

**Writing – original draft:** Hetao Huang, Biqi Pan.

**Writing – review & editing:** Jun Liu, Biqi Pan.

## Supplementary Material

Supplemental Digital Content
