# Supplementary material for: Celecoxib vs diclofenac sodium in patients with knee osteoarthritis: A protocol for systematic review and meta analysis
Source: Medicine (Baltimore). 2020 Apr 10;99(15):e19680. doi: 10.1097/MD.0000000000019680 (PMC7220482; doi:10.1097/MD.0000000000019680)
Supplement: Supplemental Digital Content [file medi-99-e19680-s001.doc]

**Search strategies in Pubmed**

#1 Search: ((((((knee osteoarthritis[MeSH Terms]) OR (knee[MeSH Terms])) OR (osteoarthritis[MeSH Terms])) OR (arthritis[MeSH Terms])) OR (knee osteoarthritis[Title/Abstract])) OR (knee[Title/Abstract])) OR (osteoarthritis[Title/Abstract])

#2 Search: (((celecoxib[MeSH Terms]) OR (Celebrex[MeSH Terms])) OR (Celebrex[Title/Abstract])) OR (celecoxib[Title/Abstract])

#3 Search: (diclofenac[MeSH Terms]) OR (diclofenac[Title/Abstract])

#4 Search: #1 and #2 and #3
